# Association of IL-10, IL-4, and IL-28B gene polymorphisms with spontaneous clearance of hepatitis C virus in a population from Rio de Janeiro

**DOI:** 10.1186/1756-0500-5-508

**Published:** 2012-09-17

**Authors:** Juliene Antonio Ramos, Rosane Silva, Luísa Hoffmann, Ana Lucia Araújo Ramos, Pedro Hernan Cabello, Turán Péter Ürményi, Cristiane Alves Villella-Nogueira, Lia Lewis-Ximenez, Edson Rondinelli

**Affiliations:** 1 Instituto de Biofísica Carlos Chagas Filho, Universidade Federal do Rio de Janeiro, Rio de Janeiro, Brazil; 2 Instituto Federal de Educação Ciência e Tecnologia do Rio de Janeiro, Rio de Janeiro, Brazil; 3 Serviço de Hepatologia, Hospital Universitário Clementino Fraga Filho, Universidade Federal do Rio de Janeiro, Rio de Janeiro, Brazil; 4 Departamento de Clínica Médica, Faculdade de Medicina, Universidade Federal do Rio de Janeiro, Rio de Janeiro, Brazil; 5 Instituto Oswaldo Cruz, Fundação Oswaldo Cruz, Rio de Janeiro, Brazil; 6 Instituto Nacional para Pesquisa Translacional em Saúde e Ambiente na Região Amazônica, Conselho Nacional de Desenvolvimento Científico e Tecnológico/MCT, Rio de Janeiro, Brazil

**Keywords:** Acute hepatitis C, Chronic hepatitis C, HCV, Spontaneous viral clearance, Cytokines, Gene polymorphism

## Abstract

**Background:**

Cytokines play an important role in the regulation of the immune response. In hepatitis C virus (HCV) infection, cytokine levels may influence the outcome of acute HCV infection. Polymorphisms in cytokine genes have been associated to different expression levels in response to infection. This study was carried out to investigate the association of several cytokine gene polymorphisms with disease outcome in HCV-infected patients.

**Findings:**

Patients with chronic or spontaneously resolved HCV infection were included in a cross-sectional study. A comparative analysis was performed between the groups regarding frequency distribution of the following cytokines’ gene polymorphisms: IL-10 (−1082 A/G; -819 T/C; -592 A/C), IL-4 (+33C/T), IFN-γ (+874 T/A), TNF-α (−238 G/A and −308 G/A) and IL-28B (rs12979860 C/T and rs8099917 T/G). Results: Eighteen patients with spontaneous viral clearance and 161 with chronic HCV infection were included. In the comparative analysis, the GG genotype of the IL-10 polymorphism -1082A/G was more frequent in patients with spontaneous viral clearance when compared to patients with chronic HCV (41.2% vs 6.2%; p = 0.001). This association was also found for the CC genotype of the IL-4 polymorphism +33C/T (72.2% vs 36.7%; p = 0.017) and the CC and TT genotypes of the IL-28B polymorphisms rs 12979860 and rs 8099917 (88.9% vs 30.3%; p < 0.001 and 88.9% vs 49.6%; p = 0.002). The IL10 (A-1082 G) and IL-28B (Crs12979860T) gene polymorphisms showed odds ratios of 12.848 and 11.077, respectively, and thus may have a greater influence on HCV spontaneous viral clearance. The IFN-γ (+874 T/A), TNF-α (−238 G/A and −308 G/A) polymorphisms did not show significant association with spontaneous viral clearance or chronicity.

**Conclusion:**

The G allele for IL-10 (−1082 A/G), the C allele for IL-4 (+3 C/T) and the C and T alleles for IL-28B (rs12979860 and rs8099917, respectively) are associated with spontaneous viral clearance in hepatitis C infection.

## Findings

### Introduction

Hepatitis C virus (HCV) infection is one of the leading causes of liver disease with approximately 180 million people chronically infected worldwide [1]. Most HCV-infected patients (70-80%) develop chronic hepatitis, which in some cases progress to liver cirrhosis (15-20%) and hepatocellular carcinoma (1-5%). Patients with spontaneous viral clearance are difficult to identify since this outcome occurs mainly in the acute phase of HCV infection, which is rarely recognized due to the lack of symptoms and signs of liver disease.

Factors related to development of persistent infection, which occurs in the great majority of cases, or to spontaneous viral clearance, are not well defined. The individual susceptibility and the outcome of HCV infection have been associated to polymorphisms of cytokines genes [2-5], and pathogenesis is related to the genotype of the virus as well as the immune response of the host [6,7].

The impact of the cytokine gene polymorphisms in the pathogenesis of HCV infection has also been investigated [8-14]. Recently, a number of studies have reported that polymorphisms near the gene for IL-28B are strongly correlated with treatment response in patients with chronic hepatitis C and also with spontaneous viral clearance [15,16]. The aim of this study was to investigate whether SNPs in genes involved in humoral and cellular immune responses as well as in genes associated with activation of the antiviral state influence the outcome of the disease towards spontaneous viral clearance or chronicity. The gene polymorphisms selected were chosen according to their described role in immune and inflammatory responses: TH1 response, IFN-γ (+874) and TNF-α (−308 and −238); TH2 response, IL-4 (+33) and IL-10 (−1082; -819 and −592); activation of the antiviral state, IL-28B rs 12979860 and rs 8099917.

### Patient characteristics

Baseline demographic of 18 patients with acute hepatitis C that progressed to spontaneous viral clearance, and 161 with chronic hepatitis C, are summarized in Table 1. Patients from both groups did not differ significantly in age (p = 0.108), gender (p = 0.145) or HCV genotype (p = 0.149). The sample size of acute hepatitis C patients is small due to the fact that patients in this phase frequently go undetected, and thus are difficult to identify. However, the statistical analysis that we performed on the data takes into account the low number of patients obtained.

**Table 1:** Demographic and virological features of patients with HCV infection

**Variable**	**Acute hepatitis C with viral clearance N = 18 (%)**	**Chronic hepatitis C N = 161 (%)**	**p**
Sex (number)			
Female	11(61.9%)	77 (55.4%)	0.133
Male	7(38.1%)	84 (44.6%)	
Age (years)	44.4 (21–73)	52.4(24–74)	0.108
Viral genotype			
1	16 (88.9%)	125 (77.6%)	0.145
other	02 (11.1%)	36 (22.4%)	

### Association of cytokine genotype and allele frequencies with the disease outcome

Patients were genotyped for IL-10 (−592C/A, -819C/T and -1082A/G) IL-4 (+33C/T), IFN-γ (+874 T/A), TNF-α (−238 G/A, -308 G/A) and IL-28B (rs12979860C/T and rs8099917T/G) gene polymorphisms. Table 2 shows the distribution of allele and genotype frequencies for the two groups. There were significant differences in the genotype distribution of IL-10 (−1082A/G) (OR 3.931, CI 1.938-7.973, p = 0.001), IL-4 (+33C/T) (OR 3.879, CI 1.655-9.089, p = 0.004), and IL-28B (rs8099917T/G and rs12979860C/T) (OR 5.021, CI 1.9-13.267 p = 0.002 and OR 5.707, CI 2.154-15.124, p < 0.001, respectively) polymorphisms. A higher frequency of GG genotype of the −1082 polymorphism of the IL-10 (41.2%) and a higher frequency of CC for +33 IL-4 polymorphism (72.2%) were observed in patients with acute hepatitis with spontaneous viral clearance than in patients with chronic hepatitis C (6.2% and 36.7% respectively; p < 0.05) It is worth mentioning that all patients with spontaneous viral clearance showed GG genotype of the −1082 polymorphism of the IL-10 together with CC genotype of the polymorphism of IL-4 +33, forming a diplotype GGCC. For the polymorphisms in the gene IL-28 gene, patients with acute hepatitis with spontaneous viral clearance had a higher frequency of TT genotype (88.9%) of the polymorphism rs8099917 and CC genotype (88.9%) of the polymorphism rs12979860. In assessing the diplotype for these two polymorphisms we found that all patients with the TT genotype of rs8099917 polymorphism also had the CC genotype polymorphism for rs12979860. Thus, the diplotype TTCC was present in 88.9% of patients who spontaneously cleared the virus.

**Table 2:** Genotype and allele frequencies of cytokine genotypes in patients with acute hepatitis C with viral clearance and patients with chronic hepatitis C

**Gene polymorphism**	**Genotype and allele**	**Acute hepatitis C with viral clearance**	**Chronic hepatitis C**	**(*p*)**
IL-10		n = 17	n = 161	
(−1082)	A/A	07 (41.2%)	106 (65.8%)	
	A/G	03 (17.6%)	45 (28%)	
	G/G	07 (41.2%)	10 (6.2%)	0.001*
	A	17 (50%)	257 (79.8%)	
	G	17 (50%)	65 (20.2%)	0.001*
(−819)	C/C	08 (47.1%)	58 (36%)	
	C/T	05 (29.4%)	60 (37.3%)	
	T/T	04 (23.5%)	43 (26.7%)	0.504
	C	21 (61.8%)	176 (54.7%)	
	T	13 (38.2%)	146 (45.3)	0.101
IL-4		n = 18	n = 147	
(+33)	C/C	13 (72.2%)	54 (36.7%)	
	C/T	04 (22.2%)	53 (36.1%)	
	T/T	01 (5.6%)	40 (27.2%)	0.017*
	C	30 (73.4%)	161 (54.8%)	
	T	06 (16.6%)	133 (45.2%)	0.004*
IFN-γ		n = 18	n = 149	
(+874)	T/T	02 (11.1%)	25 (16.4%)	
	T/A	09 (50%)	66 (44.3%)	
	A/A	07 (38.9%)	58 (38.9%)	0.804
	T	13 (36.2%)	116 (38.9%)	
	A	23 (63.8%)	182 (61.1%)	0.858
TNF-α		n = 18	n = 124	
(−308)	G/G	10 (61.9%)	75 (59.5%)	
	G/A	03 (14.3%)	27 (22.3%)	
	A/A	05 (23.8%)	22 (18.2%)	0.580
	G	23 (63.9%)	177 (71.4%)	
	A	13 (36.1%)	71 (28.6%)	0.832
(−238)	G/G	16 (88.9%)	114 (91.9%)	
	G/A	02 (11.1%)	10 (8.1%)	0.560
	G	34 (94.5%)	238 (96%)	
	A	02 (5.5%)	10 (4%)	0.388
IL-28B		n = 18	n = 119	
(rs12979860)	C/C	16 (88.9%)	36 (30.3%)	
	C/T	00 (0.0%)	61 (51.3%)	
	T/T	02 (11.1%)	22 (18.5%)	<0.001*
	C	32 (88.9%)	133 (55.9%)	
	T	04 (11.1%)	105 (44.1%)	<0.001*
		n = 18	n = 133	
(rs8099917)	T/T	16 (88.9%)	66 (49.6%)	
	T/G	00 (0.0%)	25 (18.8%)	
	G/G	02 (11.1%)	42 (31.6%)	0.002*
	T	32 (88.9%)	157 (59%)	
	G	04 (11.1%)	109 (41%)	<0.001*

* p < 0.05.

Finally, multifactorial analysis was performed to predict which polymorphisms most influenced spontaneous viral clearance (Table 3). The results show that the IL-10 (−1082) (OR 12.848 p = 0.002) and IL-28B (rs12979860) (OR 11.007 p = 0.006) gene polymorphisms have a greater influence.

**Table 3:** Multifactorial analysis of association of IL-10, IL-4 and IL-28B gene polymorphisms with spontaneous viral clearance

**Gene polymorphism**	**OR (odds ratio)**	**95% CI (confidence interval)**		***P (value)***
IL-10 (−1082)	12.848	2,478	66,606	0.002
IL-28B (rs12979860)	11.077	2.013	60.960	0.006
IL-28B (rs8099917)	5.383	0.801	36.166	0.083
IL-4 (+33)	2.467	0.633	9.610	0.193

### Discussion

This study aims to evaluate the genotype and allele frequencies of IL-10 C-592A, C-819 T and A-1082 G, IL-4 C + 33 T, IFN-γ T + 874A, and TNF-α G-238A, G-308A, a group of cytokines representative of TH1 and TH2 response, respectively, and IL28B Crs12979860T, Trs8099917G a cytokine associated with the induction of antiviral state in the cell and its association with the outcome of HCV infection in a group of Brazilian patients*.*

The allele C of the IL-4 gene is more present in the acute hepatitis C group than chronic group. To our knowledge, this is the first reported association study of IL-4 T + 33C polymorphism and spontaneous viral clearance in hepatitis C. During the course of hepatitis C, the IL-4 (+33) T allele is associated with higher expression of IL-4 gene [17] and with recurrence of infection after liver transplantation due to hepatitis C and more severe hepatic lesion [18]. The high production of IL-4 by the IL-4 (+33) TT genotype leads to a decrease in viral elimination by down regulation of the TH1 response, thereby affecting control of the viral infection. Thus, an increase in IL-4 levels may lead to an increase in liver injury [19]. On the other hand, no association was found between the IL-4 (−589) gene polymorphism and chronic hepatitis C [20].

The mechanisms responsible for the observed associations between IL-10 polymorphisms and viral infection are still unclear, but appear to be related to the level of IL-10 gene expression and the corresponding effects on the TH1 response and modulation of inflammation. SNPs in the promoter region of the IL-10 gene have been associated with low levels of IL-10 and with chronic inflammatory response in many diseases [8,9]. Individuals with IL-10 (−1082) GG genotype produce higher levels of IL-10 than GA or AA individuals [21]. Also, IL-10 is a potent inhibitor of pro-inflammatory mediators such as IL-1, TNF-α, IL-6 [10] and IFN-γ [11]. Theoretically, high production of IL-10 could facilitate viral escape by down regulating the protective TH1 response, but at the same time be beneficial to the patient due to its anti-fibrogenic properties that could lead to a slow progression of liver disease [12]. In this study, the AA genotype for IL-10 (−1082) was more frequently observed in patients with chronic hepatitis C when compared to those that resolved infection spontaneously. The higher prevalence of genotype AA, associated with low IL-10 production, may lead to a lower efficiency in the antiviral immune response. Our results suggest that a higher production of IL-10 may be associated with spontaneous viral clearance. In agreement with our results, Lio *et al*., 2003, [13] described an association between high IL-10 production and viral clearance. Thus, the predominance of IL-10 (−1082) GG genotype, associated with high IL-10 gene expression, in patients with spontaneous viral clearance may contribute to the natural history of HCV clearance. The two other IL-10 gene polymorphisms described (−819 and −592) are in linkage disequilibrium and no association with HCV infection was observed [13,21].

Gajewski *et al*., 1989, demonstrated that low levels of IFN-γ were associated with persistent HCV infection [14]. IFN-γ gene polymorphisms influence its expression, changing the levels of the cytokine, which may lead to different outcomes of the viral infection as mentioned above. In this study, no differences were found in the distribution of genotypic and allelic frequencies between patients with acute hepatitis C that cleared spontaneously and patients with chronic hepatitis C infection. These results are in agreement with those described by Pereira *et al*., 2008 [22]. However Ben-Ari *et al*., 2004 [23] reported the association of low expression genotypes of IFN-γ T + 874A polymorphism with early recurrent hepatitis C after liver transplantation. The association of the IFN-γ T + 874A polymorphism and chronicity of HCV infection is, however, still controversial.

The persistence of the virus and the response to antiviral therapy have been suggested to be associated with the production of inappropriate levels of TNF-α [24]. Nevertheless, this study did not find any differences in the distribution of genotypic and allelic frequencies, of the polymorphisms −238 and −308 of TNF-α, between patients with spontaneous viral clearance and patients with chronic hepatitis C, which is in accordance with other studies [13,22].

Several studies have demonstrated an association of SNPs in the IL-28B gene with response to treatment and spontaneous viral elimination in patients with HCV. Two of these polymorphisms, known as rs8099917 and rs12979860, have been strongly associated with non-response to treatment [15,16,25-29]. Our study shows that the genotypic frequency for the two polymorphisms in the IL-28B gene was associated with spontaneous viral clearance. Both polymorphisms, rs12979860 and rs8099917, were associated with spontaneously viral clearance in comparison with the chronically infected patients. The CC and TT genotypes (for rs12979860 and rs8099917, respectively) correspond to a positive predictive value of 89% for spontaneous viral clearance. These genotypes were also associated with spontaneous clearance of HCV in other studies [15,25,26,30]. These associations presumably are related to the ability of IL-28B to induce the antiviral state. In addition, when analyzing the influence of these polymorphisms in spontaneous viral clearance, we found a strong association with IL-10 (−1082) and IL-28B (rs12979860) gene polymorphisms, while IL-4 (+33) and IL-28B (rs8099917) gene polymorphisms had a lower influence on spontaneous viral clearance. Since the age of infection of chronic hepatitis C patients could not be determined, a limitation of the study, the ages of infection in both groups could have been different. We therefore cannot exclude the influence of different ages of infection on the observed associations. In summary, our study shows that IL-10 and IL-28B gene polymorphims have a greater, and IL-4 gene polymorphism a lower, influence on the clinical outcome of HCV infection in a Brazilian population.

### Conclusion

In conclusion: our findings show that polymorphisms in cytokine genes related to the immune response may influence the outcome of acute HCV of infection by the hepatitis C virus.

### Patients and methods

A cross-sectional study of 161 individuals with chronic HCV infection attending the Liver Clinic of the Clementino Fraga Filho University Hospital (HUCFF) were selected from 2008 to 2010 and a cohort of 18 patients with acute hepatitis C and spontaneous viral clearance were selected from the Viral Hepatitis Clinic of the Oswaldo Cruz Foundation (FIOCRUZ). This cohort of patients with acute HCV infection was previously evaluated by clinical and laboratory parameters [31]. Patients with undetected HCV RNA in three consecutive tests within 12-months of follow-up were considered as having spontaneous viral clearance. Further details were described elsewhere [31]. The inclusion criteria for chronic hepatitis C patients were: detectable anti-HCV antibodies with detectable serum, HCV RNA for 6 months of follow up, and a liver biopsy with characteristics of chronic HCV infection. Patients with clinical diagnosis of compensated liver cirrhosis (Child-Pugh A) were also included. Exclusion criteria were: HBV or HIV co-infection, alcohol ingestion, other etiologies of chronic liver disease and patients with HCV infection who were submitted to organ transplant. The age at HCV infection of chronic patients could not be determined. Written informed consent was obtained from each patient. The Research and Ethics Committee board of the HUCFF approved the study protocol.

Genomic DNA was extracted from peripheral blood using standard inorganic salting-out procedures [32]. IFN-γ +874 T/A, IL-4 +33C/T, TNF-α -238 G/A, -308 G/A, IL-10 -592C/A, -819C/T and -1082A/G and IL28B rs12979860 C/T and rs8099917T/G gene polymorphisms were genotyped using PCR and specific primers as follows: IFN-γ, F-5^′^-GGAACTTCGTTGCTCACTGGG-3^′^ and R-5^′^-CTATTACATCTACTGTGCCTTCCTG-3^′^ in a 35 cycles at 94oC for 30 seconds, 58oC for 45 seconds and 72oC for 1 min; IL-4, F- 5^′^- CAATGTAAACTCATTTTCCCTCG-3^′^ and R-5^′^-AGAAATACTGAGAGCATCACCAG-3^′^ in 35 cycles at 94°C for 30 s, 54°C for 45 s and 72oC for 1 min; IL-10, C-592A, C-819 T and A-1082 G F- 5^′^-CTCGTCGCAACCCAACTGGC-3^′^ and R-5^′^-CCTAGGTCACAGTGACGTGG-3^′^ in 35 cycles at 94o C for 30 s, 55oC for 45 s and 72o C for 1 min; TNF-α G-238A, G-308A F- 5^′^-TCCTGCATCCTGTCTGGAAGT-3^′^ and R-5^′^-AgggAgCgTCTgCTggCTgggTg-3^′^ in 35 cycles at 94o C for 30 s, 60°C for 45 s and 72°C for 1 min. Il-28B rs12979860 F- 5^′^- TCGCCAGGGCCCCTAACCTCTGC-3^′^ and R-5^′^- CGCCCAGCAGGCGCCTCTCCTA-3^′^ in 35 cycles at 94o C for 30 s, 58oC for 45 s and 72o C for 1 min and IL-28B rs8099917 F-5^′^-CCACTTCTGGAACAAATCGTC-3^′^ and R-5^′^-GATACGCTATAATTAAAGATGTGGA-3^′^ in 35 cycles at 94o C for 30 s, 54oC for 45 s and 72o C for 1 min. The amplified products were sequenced using the DYEnamic™ ET Dye Terminator Cycle Sequencing kit for MegaBACE (Amersham Pharmacia Biotech Inc.).

Statistical analysis was carried out using SPSS v15 Statistical System (Chicago, IL,USA). The Chi-square or Fisher’s exact method was used to determine deviations from Hardy-Weinberg Equilibrium and to compare genotypes, allele and phenotype frequencies. The binary logistic regression analysis was performed with the polymorphisms that showed significant differences in association analyses (P values < 0.05).

## Abbreviations

HCV: Hepatitis C virus; SNP: Single nucleotide polymorphism.

## Competing interests

The authors declare that they have no competing interests.

## Authors’ contributions

JAR contributed to the design of the study, acquired and analyzed the data, and drafted the manuscript; RS contributed to the design of the study, acquired and analyzed the data, and revised the manuscript; LH contributed to data acquirement and revision of the manuscript; ALAR contributed to cohort of patients with chronic hepatitis C; PHC contributed to statistical analysis of the data; TPU contributed to data acquirement and revision of the manuscript; CAVN contributed to cohort of patients with chronic hepatitis C; LLX contributed to the cohort of patients with acute hepatitis C; ER contributed to the design of the study, data-requirement and analysis, and drafting the manuscript. All authors have read and approved the final manuscript.

## Financial support

CNPq, CAPES, FAPERJ, CGLAB/Ministry of Health and NIH,
